# Efficacy of Highly Purified Urinary FSH versus Recombinant FSH in Chinese Women over 37 Years Undergoing Assisted Reproductive Techniques

**DOI:** 10.22074/ijfs.2015.4178

**Published:** 2015-02-07

**Authors:** Xuemei Liu, Cuifang Hao, Jinfang Wang

**Affiliations:** Reproductive Medicine Center, Yantai Yuhuangding Hospital, Affiliated Hospital of Qingdao University, Yantai, Shandong, China

**Keywords:** Urinary FSH, Recombinant FSH, IVF, ICSI

## Abstract

**Background:**

Urine derived follicle-stimulating hormone (uFSH) contains a higher proportion of acidic isoforms, whereas recombinant FSH (rFSH) contains a higher proportion of less-acidic isoforms. Less-acidic isoforms have a faster clearance, and thus a shorter half-life than the acidic FSH isoforms. The slow clearance of the acidic isoforms has a longer half-life and higher biological activity. This study was designed to determine whether uFSH or rFSH is more effective in older Chinese women undergoing assisted reproductive techniques (ART).

**Materials and Methods:**

This is a prospective, randomized, controlled cohort study. A total of 508 Chinese women over 37 years were randomized into two following study groups for their *in vitro* fertilization (IVF) or intracytoplasmic sperm injection (ICSI) cycles: i. group A (n=254) were treated with rFSH, and ii. group B (n=254) were treated with uFSH. Both groups were suppressed with a gonadotropin-releasing hormone (GnRH) analogue using a long down-regulation protocol. The main outcomes for comparison were days of stimulation, estradiol (E_2_) on the day of human chorionic gonadotropin (hCG) administration, number of oocytes collected, amount of FSH used, quantity of FSH/oocyte, endometrial thickness at hCG day, M П oocyte rate, 2PN zygote rate, grade І embryo rate, number of embryos cryopreserved, pregnancy rate, implantation rate, abortion rate and the rate of no transferable embryos.

**Results:**

Twenty two cycles including 16 cycles with poor ovarian response and six cycles with ovarian hyperstimulation syndrome were cancelled. There were 243 cycles left in each group. The patients treated with uFSH had a significantly higher 2PN zygote rate (87.4 vs. 76.6%, p<0.001), grade І embryo rate (49.8 vs. 40.8%, p<0.001) and endometrial thickness on day of hCG (11.8 mm vs. 11.2 mm, respectively, p=0.006) and a lower rate of no transferable embryos (1.2 vs. 5.3%, p=0.019) than women treated with rFSH. The other measures evaluated showed no statistically significant differences between groups (p>0.05).

**Conclusion:**

This study showed that uFSH produced a significantly higher proportion of grade І embryos than rFSH in older Chinese women and there was a significantly lower chance of no transferable embryos in uFSH cycles. The clinical efficacy of the two gonadotropins was equivalent.

## Introduction

The first birth resulting from *in vitro* fertilization (IVF) was obtained in a natural cycle (1), and since then controlled ovarian hyperstimulation (COH) has been used to generate multiple follicular growth to obtain an increased quantity of oocytes and a higher pregnancy rates. Different drug protocols have been used, such as clomiphene citrate, human menopausal gonadotropins (hMG), urine derived follicle-stimulating hormone (uFSH) and recombinant FSH (rFSH). The introduction of gonadotropin-releasing hormone (GnRH) analogues and more recently GnRH antagonists for pituitary desensitization have further enhanced pregnancy and live birth rates in IVF (2-6).

The standard down-regulation protocol with GnRH analogue plus gonadotropins for COH has gained widespread popularity because better results have been achieved in terms of number of oocytes retrieved, number of top-quality embryos obtained and pregnancy rates. Multi-follicular development is still an essential component of ovarian stimulation in IVF/intracytoplasmic sperm injection (ICSI) cycles and the quantitative aspects can be modulated by the doses of gonadotropins, the type of gonadotropin and the endocrine environment.

In recent years, ovarian stimulation protocols have focused on trying to obtain an adequate cohort of good-quality embryos instead of maximizing the number of oocytes, i.e. a shift from quantity to quality (7), especially for older patients. These older women often present with shortened early follicular phase and reduced ovarian reserve, so have a poor reproductive outcome. Several studies reported that in COH for IVF, the frequency of poor responder women is significantly higher in patients who are 40 years or older (8, 9). The number of women seeking fertility treatment at older ages is increasing in China. Thus it is very important to seek one suitable FSH product for these patients.

At present, there are two FSH products for COH, rFSH and uFSH. rFSH, produced by inserting the DNA encoding the α and β subunits of FSH into a Chinese hamster ovary cell line and containing a higher proportion of less-acidic isoforms, have been introduced for the treatment of infertility. Several studies have found that rFSH had better results in COH in terms of pregnancy rate, oocyte quality and number of oocytes retrieved compared with uFSH (10, 11). uFSH, extracted from the urine of menopausal women and containing a higher proportion of acidic isoforms, has a longer half-life and higher biological activity. It has been used successfully for many years for ovarian stimulation. Many studies compared uFSH and rFSH, but no unequivocal results have been reached (12-16). These different results may be due to different patient selection criteria, different protocols of COH, or study design. Recently, a Cochrane review discovered that differences in clinical effectiveness between the gonadotropins are small (17).

In the large number of papers published on COH protocols comparing rFSH with uFSH, there are several papers reporting studies in women with reduced ovarian reserve. Raga et al. (18) and De Placido et al. (19) reported data on small samples of young patients who were poor responders. Both studies showed that rFSH worked better than uFSH in terms of FSH amounts used, and the pregnancy rates were similar. One paper reported data on older women who were poor responders (20). The study showed that uFSH performed better in older women than rFSH when associated with the long protocol. In order to evaluate the effectiveness of uFSH and rFSH in older Chinese women, we performed a randomized controlled study comparing uFSH and rFSH in patients older than 37 years undergoing their first IVF cycle.

## Materials and Methods

### Patient Selection

This is a prospective, randomized, controlled cohort study. All patients, older than 37 years, referred to the IVF Program of Reproductive Medicine Center, Yantai Yuhuangding Hospital, Qingdao University, China, to undergo their IVF or ICSI cycle from January 2009 to December 2011, were eligible for the study. Each patient was permitted to cycle once under the study protocol. The study was reviewed and approved by Yantai Yuhuangding Hospital Ethics Committee. Informed consent was obtained from each patient before starting the trial.

Inclusion criteria were as follows: age older than 37 years, body mass index (BMI) 19-30 kg/m^2^, basal FSH <10 IU/L and estradiol (E_2_) <80 pg/ml, ≥10 antral follicles 2-10 mm in size, regular menstrual cycles of 25 to 35 days, fewer than three previous failed cycles, normal uterine cavity as assessed through hysterosalpingogram or hysteroscopy, and normal thyroid stimulating hormone (TSH) level.

Exclusion criteria were as the following: primary ovarian failure, previous poor response, history of severe ovarian hyperstimulation syndrome (OHSS), polycystic ovarian syndrome (PCOS), hydrosalpinx if it had not been surgically removed or ligated, any contraindication to pregnancy, thyroid or adrenal dysfunction, neoplasia, severe impairment of renal or hepatic function, and use of medications that might interfere with study evaluations (e.g. hormonal medication, prostaglandin inhibitors, and psychotropic agents).

A total of 508 eligible patients agreed to participate in the trial, and they were then randomized by means of a computer-generated randomization number sequence into two study groups: i. group A (n=254) were treated with highly purified uFSH (Fostimon, IBSA, Switzerland) and ii. groups B (n=254) were treated with rFSH (Gonal-F, Serono, Italy).

### Procedure

All patients of both groups underwent a standard down-regulation long protocol with GnRH analogue hormone (triptroline 0.03 mg/day, Ipsen, France). Ovarian suppression was assessed by hormonal profi les [E_2_ and luteinizing hormone (LH)] and ultrasound (US) scan of the ovaries. When suppression was confi rmed (E_2_<30 pg/mL, LH <3 IU/L, endometrial thickness ≤5 mm and no follicles of mean diameter ≥10 mm), all patients received a starting dose of 300 IU rFSH or uFSH. After 5 days, the dose of FSH was adjusted dependent on the individual response of each patient. After administration of FSH for 7 days, if there were follicles ≥14 mm in diameter, all patients in both groups would be administrated recombinant human luteinizing hormone (r-hLH; lutrophin alpha, Serono, Italy), 75 IU/day, subcutaneously until the end of ovarian stimulation. Daily gonadotropin and triptroline were continued until at least two follicles were >16 mm in average diameter. At this time, 250 μg recombinant human chorionic gonadotropin (rhCG; Vidrel, Serono, Italy) was administered.

Oocyte retrieval was performed under ultrasound (US) guidance by the transvaginal route 34-36 hours after the injection of hCG. Oocytes were fertilized either via conventional insemination or ICSI based on the semen analysis. Fertilization was assessed 16-18 hours after IVF or ICSI.

Embryos were transferred about 72 hours after fertilization. The embryos obtained were categorized into four categories, depending on their morphologic appearance (21, 22). Our center’s policy is to transfer no more than three embryos. Surplus viable embryos were cryopreserved. All transfer procedures were performed by the same physician to avoid inter-operator variability. The embryologist was blinded to the medication assignment. All pregnancies were confi rmed by a serum β-hCG 14 days after embryo transfer and US demonstration of the gestation sac 4 weeks after the transfer, at the 6^th^ week of gestation. Biochemical pregnancies alone are not included in the data analysis.

All patients received the same luteal phase support: 200 mg progesterone (Utrogest™ 200, Besins-Iscovesco, France) vaginal medication three times daily from the day of oocyte retrieval.

### Statistical Analysis

Data were analyzed with the Statistical Package for the Social Sciences (SPSS; SPSS Inc., Chicago, IL, USA) version 12.0. The endometrial thickness on day of hCG, 2PN zygote rate, grade І embryo rate and the rate of no transferable embryos (the ratio of No. of patients without transferable embryos to No. of all patients selected) were the primary outcomes. The secondary outcomes were days of stimulation, E_2_ at the day of hCG, number of oocytes collected, amount of FSH used, amount of FSH/oocyte, M П oocyte rate, number of embryos cryopreserved, pregnancy rate, implantation rate and abortion rate. Data were expressed as mean ± SD or percentages. Differences between groups of continuous variables were analyzed with T test and the chi-square test was used to assess differences in proportions. Value of p<0.05 was considered statistically significant.

## Results

A total of 508 assisted reproductive techniques (ART) cycles were analyzed in the present investigation. No significant differences were observed among the two study groups in terms of age, BMI, infertility duration, basal FSH levels, and causes of infertility (Table 1).

A total of 22 cycles were cancelled. Among those, there were 16 cases (nine cases in group A and seven cases in group B) owing to poor ovarian response (no follicles of diameter ≥10 mm after administration of FSH for 7 days), and there were 6 cases (two cases in group A and four cases in group B) because of OHSS [with abdominal distention, nausea, vomiting, ascites, hydrothorax, hematocrit (HCT) >41%, pericardial effusion, white blood cell (WBC) >10000/mm^3^, oliguria or anuria, etc.]. There were 243 cycles left in each group. There were 3 cycles without transferable embryos in group A and 13 cycles in group B.

Table 2 shows comparisons between the two groups in both uFSH and rFSH protocols regarding stimulation characteristics, oocyte quality, embryo quality and treatment outcome. No significant differences between groups were found for days of stimulation, E_2_ at the day of hCG, number of oocytes collected and M П oocyte rate. The total of FSH used, amount of FSH/oocyte and number of embryos cryopreserved were higher in the uFSH group than those in rFSH group, but the differences are not statistically significant.

The endometrium on day of hCG was significantly thicker in the uFSH group than in rFSH group (11.8 mm vs. 11.2 mm, respectively, p=0.006). Regarding embryo quality, the proportion of grade І embryos on day 3 was significantly higher in the uFSH group than that in rFSH group (49.8 vs. 40.8%, respectively, p<0.0001). The proportion of 2PN zygotes (normal fertilization) to all the zygotes present on day 1 in uFSH group was also found to be significantly higher than that in the rFSH groups (87.4 vs. 76.6%, p<0.0001).

The rates of clinical pregnancy and implantation rate were not significantly different between the two groups. However, there was a trend to a lower abortion rate in the uFSH group which might be noteworthy, even if it did not reach statistical significance in the present study (23.5 in uFSH group vs. 29.1% in rFSH group). Moreover, the rate of no transferable embryos was significantly lower in the uFSH group than that in the rFSH group (1.2 vs. 5.3%, respectively, p=0.019).

**Table 1:** Demographic characteristics of 508 patients


	uFSH	rFSH	P value

**Age (Y)**	39.1 ± 1.7	38.9 ± 1.7	NS
**Body mass index (kg/m^2^)**	24.4 ± 3.1	24.2 ± 3.2	NS
**Infertility duration (Y)**	7.6 ± 4.9	7.4 ± 4.8	NS
**Basal FSH (IU/L)**	7.1 ± 2.3	7.3 ± 3.2	NS
**Cause of sterility**			
Tubal factor	51.9%	53.5%	NS
Male factor	4.5%	4.1%	NS
Mixed	35.0%	31.7%	NS
Endometriosis	4.5%	5.4%	NS
Unexplained	4.1%	5.3%	NS


Values are mean ± SD or percentages, NS; Not significant difference among groups (p>0.05), FSH; Follicle-stimulating hor-mone, uFSH: Urine derived follicle-stimulating hormone and rFSH: Recombinant follicle-stimulating hormone.

**Table 2:** Comparison between uFSH and rFSH in women over 37 years old


	uFSH	rFSH	P value

**No. of cycles**	243	243	
**Days of stimulation**	8.8 ± 1.2	8.8 ± 1.3	NS
**Total FSH used (IU)**	2633.1 ± 407.0	2549.0 ± 578.2	NS
**E_2_ at hCG day (pg/mL)**	2749.4 ± 1259.3	2641.1 ± 1408.6	NS
**Endometrial thickness at hCG day (mm)**	11.8 ± 2.3	11.2 ± 2.4	0.006
**Oocytes retrieved**	8.1 ± 4.9	8.9 ± 4.8	NS
**FSH used/oocyte (IU/oocyte)**	444.7 ± 282.4	442.8 ± 315.7	NS
**M П oocytes/total number of oocytes**	78.8%	79.7%	NS
**2PN zygote rate**	87.4%*	76.6%*	<0.0001
**Grade І embryos/all embryos at day 3**	49.8%*	40.8%*	<0.0001
**NO. of embryos cryopreserved**	486	402	0.68
**Rate of no transferable embryos**	1.2%*	5.3%*	0.019
**Clinical pregnancy rate**	47.9%	47.8%	NS
**Implantation rate**	26.6%	30.1%	NS
**Abortion rate**	23.5%	29.1%	NS


Values are mean ± SD or percentages, NS; Not significant difference among groups (p>0.05), FSH; Follicle-stimulating hor - mone, uFSH; Urine derived follicle-stimulating hormone, rFSH; Recombinant follicle-stimulating hormone, E_2_; Estradiol , hCG; Human chorionic gonadotropin and *; Values are significantly different among groups (p<0.05).

## Discussion

Numerous studies have compared rFSH and urinary gonadotropins in terms of clinical efficacy and efficiency, but this remains a controversial area (10-14). A recent meta-analysis (23) showed that rFSH worked better than uFSH in terms of clinical efficacy, but another (24) showed the opposite. There is often considerable homogeneity of patients within studies in terms of age, race, etc., but considerable differences have been found between different study protocols in terms of dosing, route of administration and different uFSH products.

In the study reported here, we used a prospective, randomized controlled trial (RCT) design and selected 508 Chinese women over 37 years as subjects. They had similar demographic characteristics, including age, BMI, infertility duration, basal FSH levels and causes of infertility, and were treated with the same protocol.

In this RCT, we found that the 2PN zygote rate, grade І embryo rate and endometrial thickness at hCG day in uFSH group were significantly higher than those in the rFSH group, and the rate of no transferable embryos was significantly lower. The other endpoints, including days of stimulation, the total of FSH used, rate of FSH/oocyte, E_2_ at the day of hCG, oocyte number, M П oocyte rate, number of embryos cryopreserved, clinical pregnancy and implantation rate showed no statistically significant differences between the two groups.

In older women, the early follicular phase is shortened (25). It may predict ovarian ageing and induce lower clinical and viable pregnancy rates.

With advancing age, acidic isoforms (highly glycosylated) of FSH is progressively increasing (26), which induces a slower progression to antral phase, may in part balance the fastened progression of follicles in older patients. Moreover, acidic FSH isoforms are produced during the follicular and luteal phases (when the estradiol level is low), whereas less-acidic FSH isoforms are produced during the mid-cycle (when the estradiol level is high) during a normal menstrual cycle (27, 28).

uFSH contains a higher proportion of acidic isoforms, but rFSH contains a higher proportion of less-acidic isoforms (29). Less-acidic isoforms have a faster clearance and thus a shorter half-life than the acidic FSH isoforms (30, 31). The slow clearance of the acidic isoforms has a longer half-life and stronger stimulation (32). At start of stimulation with acidic FSH (uFSH), there were fewer follicles developing and at a slower growth rate. The follicles stimulated with acidic FSH require 5 days to reach the dimensions recorded at days 3 with least acidic FSH (31).

The slower growth rate in pre-antral phase will induce a longer pre-antral phase. It is mandatory for good quality oocytes and priming the oocyte for a correct reproductive function by DNA imprinting, genetic synthesis and protein synthesis. On the other hand, acidic FSH has higher follicular threshold and only "good" follicles are stimulated (33). Therefore, uFSH, due to its unique content in highly glycosylated FSH, stimulates the follicles in a more physiologic way in older women. In COH cycles, as compared to rFSH, a lower number of follicles are recruited and the initial slower development induces better quality of oocytes and embryos, and so have higher 2PN zygote rate and grade І embryo rate. In addition, uFSH improved the development of endometrium in this study.

Other trials, described hereinafter, have examined the clinical efficacy specifically of Fostimon versus Gonal-F. In recent studies, investigators found that patients treated with uFSH used a significantly less medication than those treated with rFSH, and that they are equivalent in terms of clinical efficacy in older women (20). Selman et al. (34) found that the grade І embryo score was significantly higher in the uFSH group than the rFSH group, even though no statistically significant difference in pregnancy rate was registered. A recently published study found a higher pregnancy rate in patients randomized to the combination of uFSH and rFSH when compared with those randomized to rFSH alone (35). Overall, these studies suggest that uFSH is as effective, efficient, and safe for clinical use as rFSH. Many studies show that exogenous LH administration could lead to more optimal follicular development and a higher pregnancy rate in women aged over 35 years (36-38). LH is important in regulating steroidogenesis throughout follicular development and adequate LH is particularly important for oocyte maturation (39), so we supplemented LH in both groups in our study.

Our study showed that uFSH performed better than rFSH in terms of 2PN zygote rate and grade 1 embryo rate in older Chinese women, and the rate of no transferable embryos was reduced. These results may be explained by the fact that uFSH contains relatively higher acidic isoforms which show a decreased clearance rate, and may improve oocyte and embryo quality.

It is well recognized that for women in late reproductive age, oocyte and embryo quality decrease, and lead to a low on-going pregnancy rate. Our study suggested that uFSH might promote better oocyte and embryo quality compared to recombinant gonadotropins. An explanation may be due to the reason that it contains a higher proportion of acidic isoforms and more suitable for the older women. Further studies are needed to confirm these data and to establish the best protocol for different groups of patients.

## Conclusion

This study was designed to determine whether uFSH or rFSH is more effective in older Chinese women undergoing ART. We found that 2PN zygote rate, grade І embryo rate and endometrial thickness were significantly higher in uFSH group, and the rate of no transferable embryos was significantly lower. The study suggested that uFSH might promote better oocyte and embryo quality and endometrial thickness compared with recombinant gonadotropins in older Chinese women.
